# Application of multi-gene genetic programming to the prognosis prediction of COVID-19 using routine hematological variables

**DOI:** 10.1038/s41598-024-52529-y

**Published:** 2024-01-23

**Authors:** Hamid Reza Niazkar, Jalil Moshari, Abdoljavad Khajavi, Mohammad Ghorbani, Majid Niazkar, Aida Negari

**Affiliations:** 1 Gonabad University of Medical Sciences, Gonabad, Iran; 2 Breast Diseases Research Center, Shiraz University of Medical Sciences, Shiraz, Iran; 3 Pediatric Department, School of Medicine, Gonabad University of Medical Sciences, Gonabad, Iran; 4 Community Medicine Department, School of Medicine, Gonabad University of Medical Sciences, Gonabad, Iran; 5 Laboratory hematology and Transfusion medicine, Department of Medical Laboratory Sciences, Faculty of Allied Medicine, Gonabad University of Medical Sciences, Gonabad, Iran; 6 Faculty of Engineering, Free University of Bozen-Bolzano, Piazza Università 5, 39100 Bolzano, Italy

**Keywords:** Infectious diseases, Viral infection

## Abstract

Identifying patients who may develop severe COVID-19 has been of interest to clinical physicians since it facilitates personalized treatment and optimizes the allocation of medical resources. In this study, multi-gene genetic programming (MGGP), as an advanced artificial intelligence (AI) tool, was used to determine the importance of laboratory predictors in the prognosis of COVID-19 patients. The present retrospective study was conducted on 1455 patients with COVID-19 (727 males and 728 females), who were admitted to Allameh Behlool Gonabadi Hospital, Gonabad, Iran in 2020–2021. For each patient, the demographic characteristics, common laboratory tests at the time of admission, duration of hospitalization, admission to the intensive care unit (ICU), and mortality were collected through the electronic information system of the hospital. Then, the data were normalized and randomly divided into training and test data. Furthermore, mathematical prediction models were developed by MGGP for each gender. Finally, a sensitivity analysis was performed to determine the significance of input parameters on the COVID-19 prognosis. Based on the achieved results, MGGP is able to predict the mortality of COVID-19 patients with an accuracy of 60–92%, the duration of hospital stay with an accuracy of 53–65%, and admission to the ICU with an accuracy of 76–91%, using common hematological tests at the time of admission. Also, sensitivity analysis indicated that blood urea nitrogen (BUN) and aspartate aminotransferase (AST) play key roles in the prognosis of COVID-19 patients. AI techniques, such as MGGP, can be used in the triage and prognosis prediction of COVID-19 patients. In addition, due to the sensitivity of BUN and AST in the estimation models, further studies on the role of the mentioned parameters in the pathophysiology of COVID-19 are recommended.

## Introduction

In December 2019, for the first time in Wuhan, China, a new type of coronavirus was identified in patients with pneumonia of unknown cause. Later, this novel beta-coronavirus was named severe acute respiratory syndrome coronavirus 2 (SARS-Cov-2)^1,2^. The mysterious nature of the coronavirus disease 2019 (COVID-19), and its rapid respiratory human-to-human transmission brought about a wild outbreak, which was announced to be a pandemic by World Health Organization (WHO) in March 2020. Based on the latest statistics as of November 2023, the emerging coronavirus has traveled worldwide and infected more than six hundred ninety million people, leading to the death of more than six million nine hundred thousand individuals^3^.

The COVID-19 patients present unspecific signs and symptoms including, but not limited to, fever, cough, sore throat, dyspnea, myalgia, and headache. In addition, most patients with COVID-19 may be asymptomatic, or experience disease as mild as simple flu, whereas some patients may develop a serious disease, requiring hospitalization, or even leading to death^4^. Currently, the nasopharyngeal reverse transcription-polymerase chain reaction (RT-PCR) swab test is considered the test of choice for the diagnosis of COVID-19^4^. Furthermore, commonly reported laboratory changes such as lymphopenia, and increased inflammation indices along with the typical computed tomography (CT) characteristics of COVID-19, such as peripheral ground-glass opacities, can help diagnose COVID-19^5^.

The COVID-19 pandemic prompts a healthcare crisis. In this regard, during the outbreaks, patients’ rush to the hospital may result in the insufficient hospital or intensive care unit (ICU) beds, mechanical ventilators, drugs, and other necessary equipment. This unarguably raises the importance of medical resource allocation^6^. To be more specific, the appropriate allocation of hospital beds, ICU beds, mechanical ventilators, and other limited medical tools and equipment is one of the challenges for healthcare decision-makers in combatting the COVID-19 pandemic. Moreover, predicting the prognosis of COVID-19 patients can help allocate medical resources and provide appropriate supportive care^7^.

Previous studies have suggested various hematologic factors for the prognosis prediction of COVID-19 patients^5,8,9^. Despite vaccination programs and various applications of machine learning methods for the prognosis prediction of COVID-19, the significance of each proposed prognostic factor is not clear yet. Moreover, the quest for prognosis prediction of COVID-19 is still ongoing. In this regard, the current study aimed to predict the prognosis of COVID-19 patients using multi-gene genetic programming (MGGP) incorporating conventional laboratory tests at the time of admission. In this regard, MGGP is a variant of genetic programming (GP) inspired by Darwinian evolution, a natural process describing how a few individuals with specific characteristics (i.e., genes) survive in a population. As an improved version of GP, MGGP provides an opportunity to capture highly nonlinear complex relationships governing a physical system or a phenomenon as it permits the development of highly nonlinear prediction models. So far, MGGP has been applied for various purposes in the medicine. For instance, Sattar and colleagues^10^, applied MGGP for the diagnosis of lung cancer at early stages using lung cancer related mutated genes, which yielded to an accuracy of 95.67%^10^. Similarly, Kamrul Hasan and colleagues, utilized MGGP models for early prediction of the breast cancer^2^. Furthermore, Niazkar et al.^11^, applied MGGP to predict the incidence of COVID-19 in seven countries. They observed that despite considerable fluctuations in daily cases, MGGP is still capable of estimation the daily cases with promising accuracy^12^. In addition, according to the literature, it is the first time that MGGP has been used to predict the duration of hospital stay, ICU admission, and mortality of COVID-19 patients. Such estimations can facilitate the triage, and personalized treatment and optimize the allocation of medical resources.

## Materials and methods

### Study design and data collection

This retrospective study was conducted on COVID-19 patients hospitalized in Allameh Behlool Gonabadi from 2020 to 2021. It was approved by the medical ethics committee of the Gonabad University of Medical Sciences (ethic code: IR.GMU.REC.1400.060). All methods were performed in accordance with the relevant guidelines and regulations. Also, the informed consent was obtained from all subjects and/or their legal guardian(s).

The inclusion criteria were a positive nasopharyngeal RT-PCR test for COVID-19 and admission to infectious disease or internal medicine wards. In addition, exclusion criteria were pregnant women, infants, neonates, and pediatrics with COVID-19, discharge/leave against medical advice, transfer or referral to other hospitals, patients with missing data, and those with pre-existing chronic medical diseases. The list of hospitalized COVID-19 patients was collected from the hospital information registration system. The collected information includes age, sex, and the prognosis of patients (duration of hospital stay, admission to ICU, mortality). Based on the inclusion criteria, 2660 out of 3243 patients were enrolled in the study. After applying exclusion criteria, 2342 patients were selected.

The common laboratory tests performed at the time of admission were retrieved from the hospital’s electronic registration system using the national code of the hospitalized patients. These tests include a complete blood count (CBC) (white blood cell count, red blood cell count, hemoglobin level, platelet count, absolute neutrophil count, and absolute lymphocyte count), coagulation factors including partial thromboplastin time (PTT), prothrombin time (PT), inflammatory indices such as c-reactive protein (CRP), erythrocyte sedimentation rate (ESR), and biochemical factors such as creatinine, blood urea nitrogen (BUN), aspartate aminotransferase (AST), alanine aminotransferase (ALT) and alkaline phosphatase level . Finally, after excluding patients with missing data, 1455 patients (727 male, 728 female) were chosen for the study.

### Multi-gene genetic programming

Genetic programming^13^ is an advanced genetic algorithm (GA), which is capable of solving complex problems and was inspired by the process of natural selection^14^. In addition, GP can develop reliable time- and cost-effective applications by exploiting GA as a search engine and optimization algorithm^15^. In this regard, various variants of GP have been proposed in the literature including MGGP. Nevertheless, most GP versions follow a similar tree-based structure to attain a relation between input and output variables^16^.

**Figure 1:**
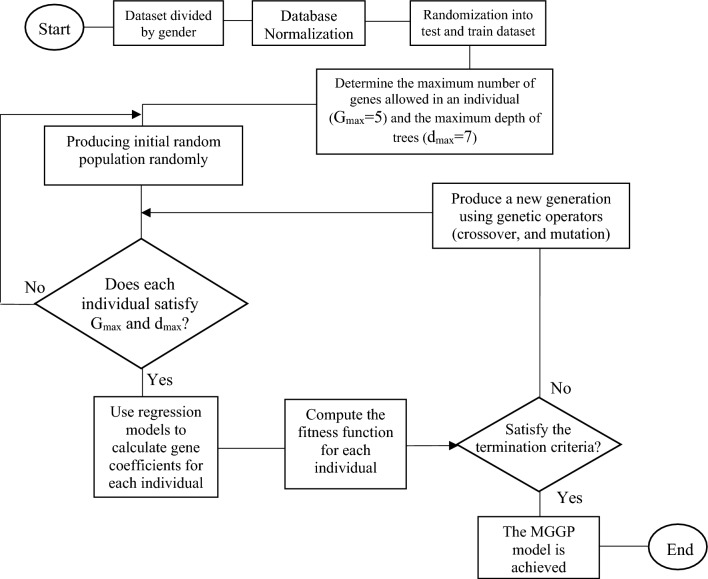
Flowchart of MGGP model for developing estimation models.

Generally, MGGP entails four steps including initialization, selection, reproduction, and termination, which are depicted in Fig. 1. As shown, in the initialization step, MGGP produces a random population comprising functions and terminals. The generated population is modified by GA operators to achieve the best relation between input and output variables. The functions frequently used by MGGP include the four basic operations of mathematics, Boolean operators, the root function, exponential and logarithmic functions, and various trigonometric functions^17^. Since the initial population is created using random combinations of different functions and constant coefficients, it is not required to know the nature of the prediction model functions in advance of developing a prediction model^9^.

Each individual in MGGP is a predictive model, which can consist of one or more than one gene (trees). Additionally, the fitness function is calculated for each MGGP individual to rank its performance in comparison to other individuals existing in a specific population^18^. If individuals created in the original population do not provide the desired fitness function, a new population is produced. In the selection stage, individuals with a better fitness function are selected and used to create a new population in the reproduction stage. As illustrated in Fig. 1, the reproduction phase in MGGP is performed in conjunction with GA operators (selection, crossover, and mutation)^19^. In the final stage, the desired conditions are defined and evaluated as an acceptable error threshold or the maximum number of generations produced. Technically, MGGP continues to execute until the termination criterion is met. Therefore, a prediction function with optimal accuracy will be obtained in the final stage of MGGP^14^.

In MGGP, the maximum number of genes allowed in an individual (G_max_) and the maximum depth of trees (d_max_) are two important controlling parameters^20^. Needless to say, selecting values of these two parameters is a trade-off. In other words, the development of a more accurate model may be possible by increasing the values of these two parameters. However, achieving such accuracy may lead to a more complex model that inevitably requires more computational efforts . In this study, G_max_ and d_max_ were set at five and seven, respectively. Furthermore, an open-source code of MGGP in MATLAB software, which has been adapted from reputable sources, was used^21^. The controlling parameters applied for the development of MGGP are following the previously conducted study.

In the present study prognosis prediction models were developed for each gender separately. The reason for the dividing genders was to decrease the input variables and increase the accuracy of obtained models as much as possible. Input variables are introduced to the software as shown in Table 1.

**Table 1:** Input variables of the current study.

Parameter	Variable	Parameter	Variable
X_1_	Age	X_9_	PTT
X_2_	White blood cells	X_10_	CRP
X_3_	Red blood cells	X_11_	ESR
X_4_	Hemoglobin	X_12_	ALP
X_5_	Absolute neutrophil count	X_13_	AST
X_6_	Absolute lymphocyte count	X_14_	ALT
X_7_	Platelet	X_15_	Creatinine
X_8_	PT	X_16_	BUN

The collected database was normalized before analysis in MGGP, based on the following equation^22^:

1 $$\overline{f }=({f}_{i}-{f}_{min})/({f}_{max}-{f}_{{\text{min}}})$$

In the above equation $\overline{f }$ is the normal value of data, ${f}_{max}$ is the maximum value of data, ${f}_{{\text{min}}}$ is the minimum value of data, and ${f}_{i}$ is the ith data.

The normalized data (727 males and 728 females) were randomly divided into the train (two-thirds of the total data) and test dataset (one-third of the total data). Therefore, out of 727 male patients, 487 were selected as training data and 240 were selected as test data. Also, out of 728 female patients, 484 were selected as training data and 244 were selected as test data. In addition, using the training dataset, prediction models were calibrated for each outcome parameter (mortality, hospitalization in ICU, and length of hospital stay) for each sex separately.

Since MGGP is a search-based AI method, each implementation of this program may result in a unique mathematical model. Based on the current literature, after at least 50 times of program execution, the best (most accurate) model can be considered as a result of this AI tool^22^. Thus, in the present study, for each of the output parameters and each gender, the MGGP program has been executed at least 50 times to achieve the most accurate model.

### Evaluation of prediction models

The accuracy of the prediction models obtained by MGGP was evaluated using the following three equations^22^:

2 $$RMSE=\sqrt{\frac{1}{N}\sum_{i=1}^{N}{({HS}_{observed,i}-{HS}_{estimated,i})}^{2}}$$

3 $${\text{MARE}}=\frac{1}{N}\sum_{i=1}^{N}\left|\frac{{HS}_{observed,i}-{HS}_{estimated,i}}{{HS}_{observed,i}}\right|$$

4 $${{{\text{R}}}^{2}=\left\{\frac{\sum_{{\text{i}}=1}^{{\text{N}}}[({HS}_{observed,i}-\frac{1}{N}\sum_{i=1}^{N}{HS}_{observed,i})({HS}_{estimated,i}-\frac{1}{N}\sum_{i=1}^{N}{HS}_{estimated,i})]}{\sqrt{\sum_{{\text{i}}=1}^{{\text{N}}}[{({HS}_{observed,i}-\frac{1}{N}\sum_{i=1}^{N}{HS}_{observed,i})}^{2}{({HS}_{estimated,i}-\frac{1}{N}\sum_{i=1}^{N}{HS}_{estimated,i})}^{2}]}}\right\}}^{2}$$

In the above equations, ${HS}_{observed,i}$ is the observational hospital stay for the $i$ patient in the hospital, ${HS}_{estimated,i}$ is the estimated hospital stay for the $i$ patient in the hospital, N is the number of patients, RMSE is the root-mean-square error, MARE is the mean absolute relative error, and R^2^ is the determination coefficient. Based on the metric definitions, the lower the RMSE and MARE values are, and the closer the R^2^ value is to one, the more accurate the prediction model will be.

### Sensitivity analysis

In this study, in a bid to determine the significance of the input parameters in each of the prediction models, a sensitivity analysis was performed based on the following equation^22^:

5 $${SA}_{i}=\frac{{HS}_{max}\left({x}_{i}\right)-{HS}_{min}\left({x}_{i}\right)}{\sum_{i=1}^{N}\left[{HS}_{max}\left({x}_{i}\right)-{HS}_{min}\left({x}_{i}\right)\right]}\times 100$$

where ${HS}_{max}\left({x}_{i}\right)$ and ${HS}_{min}\left({x}_{i}\right)$ are the maximum and minimum hospital stay durations, respectively, and ${SA}_{i}$ is the sensitivity analysis percentage of the ith parameter. Also, a higher value of ${SA}_{i}$ denotes that the corresponding parameter has a higher impact on the outcome.

### Ethic approval

The present study was approved by ethic committee of Gonabad University of Medical Sciences with the code IR.GMU.REC.1400.060.

## Results

The present study was conducted on 1455 COVID-19 patients (727 men, 728 women), of which 147 patients were admitted to the ICU. Also, out of 1455 patients, 1250 patients recovered from COVID-19 and 205 patients died. The descriptive analysis of studied variables is presented in Table 2.

**Table 2:** The descriptive analysis of variables in the present study.

Variable	Min	Max	Average	Standard Deviation
Age	16	104	62.10	18.49
White blood cells	300	198,000	7733	7981.64
Red blood cells	2.49	7.21	4.69	0.69
Hemoglobin	3.70	21.70	13.41	2.17
Absolute neutrophil count	0	112,000	5816.20	5122.15
Absolute lymphocyte count	100	129,000	1452.7	4017.38
Platelet	4000	238,000	211,130	106,053
PT	10.5	134	14.07	5.92
PTT	12.3	130	35.5	11.56
CRP	0	4	1.34	1.09
ALP	4	4880	199.49	288.08
ESR	0	577	40.27	30.58
AST	7	4740	65.45	174.84
ALT	3	3120	47.04	107.07
Cr	0.07	56	1.31	1.9
Bun	0	330	21.62	18.14
Hospital stay	1	70	7.32	6.55

The MGGP-based prediction models were developed for each gender and each output variable separately. These explicit models are presented in the followings:

### Prediction of mortality

The obtained prediction model for the outcome (mortality) of female COVID-19 patients ${({\text{Outcome}}}_{f})$ is as follows:

$${{\text{Outcome}}}_{f}=\left\{\begin{array}{cc}0& A\le 0.8\\ 1& {\text{A}}>0.8\end{array}\right.,$$

6 $$A=35.83\times {\text{cos}}\left({x}_{13}\times {x}_{14}\right)\times {\text{cos}}\left({x}_{16}\right)+47.14\times \left({x}_{9}-2\times {x}_{16}\right)+115.4\times {x}_{14}\times \left({x}_{1}-{x}_{14}\right)\times \left({x}_{6}-{x}_{9}\right)\times \left({x}_{9}-{x}_{14}\right)-34.82$$

where ${{\text{Outcome}}}_{f}=0$ means death and ${{\text{Outcome}}}_{f}=1$ means recovery of the patient.

Table 3 presents the accuracy and error percentage of the MGGP-based prediction models for the train and test dataset. As shown, Eq. (2) has only failed in the outcome prediction of 35 data out of 484 training data, and performed with an accuracy of 92.77%. Moreover, in the test dataset, it failed in the outcome estimation of 66 data out of 244 data and has gained an accuracy of 72.95%.

**Table 3:** Accuracy and error percentage of the MGGP-based prediction models for train and test dataset.

Outcome	Gender	Metrics	Train Dataset	Test Dataset
Mortality	Female	Accuracy	92.77	72.95
		Error Percentage	7.23	27.05
	Male	Accuracy	79.88	60.42
		Error Percentage	20.12	39.58
ICU admission	Female	Accuracy	91.32	76.64
		Error Percentage	8.68	23.36
	Male	Accuracy	90.76	80.83
		Error Percentage	9.24	19.17
Hospital stay	Female	Accuracy	58.68	53.69
		Error Percentage	41.32	46.31
	Male	Accuracy	65.7	61.25
		Error Percentage	34.3	38.75

**Figure 2:**
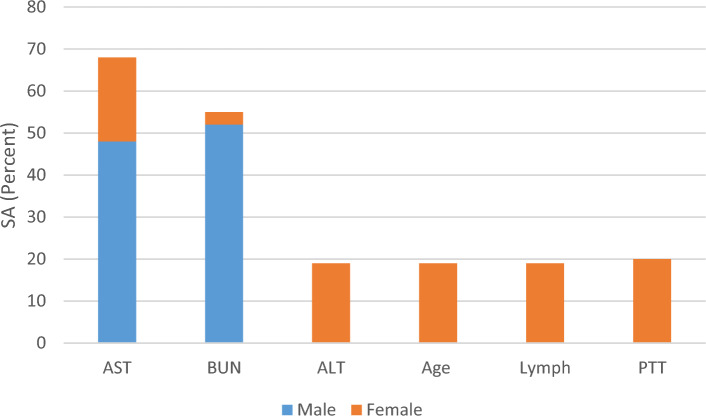
Sensitivity analysis results for the MGGP-based models predicting the mortality of the COVID-19 patients.

Figure 2 depicts the results of the sensitivity analysis for estimating the mortality of COVID-19 patients. As shown, it revealed that among the studied parameters, age, absolute lymphocyte count, PTT and AST with equal percentage, and then the ALT and BUN have the most significance in the prediction of mortality of female COVID-19 patients.

The prediction model achieved by MGGP for the outcome (mortality) of male COVID-19 patients ${({\text{Outcome}}}_{m})$ is as follows:

$${{\text{Outcome}}}_{m}=\left\{\begin{array}{cc}1& B\le -0.003\\ 0& {\text{B}}>-0.003\end{array}\right.,$$

7 $$B=-84.72\times square\left({x}_{13}\times {x}_{16}\right)-23.9\times square\left({x}_{10}\left({x}_{5}-{x}_{13}\right)\right)+84.72\times {x}_{13}\times square\left({x}_{16}\right)+1.03$$

where ${{\text{Outcome}}}_{f}=0$ and ${{\text{Outcome}}}_{f}=1$ indicate the death and recovery of a patient, respectively.

According to Table 3, Eq. (7) has failed in predicting the outcome of 98 out of 487 training data, which demonstrates that it reaches an accuracy of 79.88%. In addition, for the test data, yielded wrong predictions for 95 out of 240 data and has gained an accuracy of 60.42% (Table 3). Based on Fig. 2, the results of the sensitivity analysis imply that, among the input variables, BUN and AST have the most significant influence on the prediction of the mortality of male COVID-19 patients.

### Prediction of ICU admission

The MGGP-based prediction model for estimating whether female COVID-19 patients require an ICU admission is presented in Eq. (8):

$${ICU}_{f}=\left\{\begin{array}{cc}0& C\le 0.039\\ 1& {\text{C}}>0.039\end{array}\right.,$$

8 $$C=1.191\times {x}_{1}\times \left({{x}_{9}}^{2}+{x}_{13}-{x}_{15}\right)-0.0341$$

In the above relation, ${ICU}_{f}=0$ denotes no ICU admission, whereas ${ICU}_{f}=1$ means that an ICU admission is required.

As shown in Table 3, the prediction model given in Eq. (8) reported the wrong prediction of the outcome for 42 out of 484 training data, and performed with an accuracy of 91.32%. Also, it has gained an accuracy of 76.64%, which corresponds to failure in the outcome prediction of 57 out of 244 test data.

**Figure 3:**
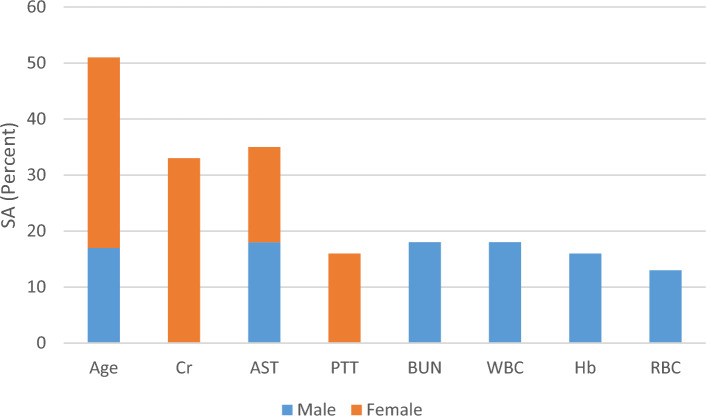
Sensitivity analysis results for the MGGP-based models predicting the ICU admission of the COVID-19 patients.

Figure 3 illustrates ${SA}_{i}$ for MGGP-based models developed for forecasting ICU admission. As shown, the sensitivity analysis revealed that age, creatinine, AST level, and PTT have the highest impacts on the ICU admission of female COVID-19 patients.

In addition, the ICU admission prediction model for male COVID-19 patients is as follows:

$${ICU}_{m}=\left\{\begin{array}{cc}0& D\le 0.25\\ 1& {\text{D}}>0.25\end{array}\right.,$$

9 $${ICU}_{m}=37.98\times {x}_{5}\times {\text{tanh}}\left({x}_{16}\right)-\frac{0.08697\times {x}_{13}}{{x}_{2}+{x}_{5}}-2.824\times {x}_{13}-21.85\times {x}_{16}\times \left({x}_{13}+{x}_{16}\right)\times \left({x}_{2}+2\times {x}_{5}\right)-18.99\times {x}_{1}\times {x}_{5}\times ({x}_{3}-{x}_{4})-0.04006$$

where ${ICU}_{f}=0$ means no ICU admission, whereas ${ICU}_{f}=1$ denotes an ICU admission.

According to Table 3, Eq. (9) has failed in the outcome prediction of 45 out of 487 training data, and performed with an accuracy of 90.76%. Furthermore, in test data, the achieved prediction model failed in the outcome prediction of 46 out of 240 data and has gained an accuracy of 80.83%. Also, Fig. 3 demonstrates that the sensitivity analysis showed that ICU admission of male COVID-19 patients is mostly affected by age, absolute leukocyte count, AST, and BUN levels followed by hemoglobin, red blood cell count, and absolute neutrophil count.

### Prediction of hospital stay

The achieved prediction model for the duration of hospital stay in female COVID-19 patients ${({\text{HS}}}_{f})$ is as follows:

10 $${HS}_{f}=0.1461\times {x}_{9}-0.1461\times {x}_{3}-\frac{22.71\times {x}_{3}\times {x}_{16}\times \left({x}_{3}-{x}_{9}\right)}{{x}_{9}+{x}_{3}\times {x}_{9}-8.723}-\frac{0.003789\times {x}_{2}\times {x}_{3}\times \left({x}_{7}-{x}_{9}\right) }{{x}_{2}-{x}_{11}+{x}_{6}\times {x}_{11}}+0.09921$$

The results obtained by the above model are denormalized for comparison with the observed data. The calculated RMSE, MARE and R^2^ parameters for this estimation model in the training data are 5.17, 0.62 and 0.23, respectively. Also, the calculated RMSE, MARE and R^2^ parameters in the test data are 11.62, 1.17 and 0.0006, respectively (Table 4). In a bid to evaluate the accuracy and the performance of the obtained model (Eq. 10), the duration of hospital stay was divided into three periods less than one week, more than one week and less than two weeks, and more than two weeks. This prediction model accurately predicted the hospital stay of 284 out of 484 patients in the training data, achieving an accuracy of 58.68%. Also as shown in Table 3, in test data, the developed prediction model gained an accuracy of 53.69%, accurately predicting the hospital stay of 131 out of 244 patients.

**Table 4:** Evaluation criteria of hospital stay prediction models.

Gender	Criteria	Train Dataset	Test Dataset
Female	RMSE	5.17	11.62
	MARE	0.62	1.17
	R^2^	0.23	0.0006
Male	RMSE	4.59	9.52
	MARE	0.77	1.26
	R^2^	0.09	0.0025

**Figure 4:**
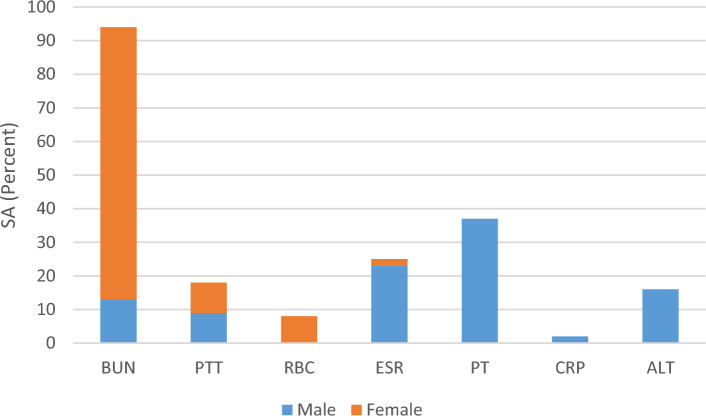
Sensitivity analysis results for the MGGP-based models predicting the hospital Stay duration of the COVID-19 patients.

Furthermore, sensitivity analysis indicated that among the input variables, BUN, PTT, red blood cell count and ESR have the most significance in the hospital stay prediction of female COVID-19 patients (Fig. 4).

The achieved prediction model for the hospital stay in male COVID-19 patients ${({\text{HS}}}_{m})$ is as follows:

11 $${HS}_{m}=-\frac{\frac{7.214\times \left({x}_{9}+\frac{{x}_{7}}{{x}_{14}}-1.473\right)}{{10}^{5}}}{{x}_{1}-{x}_{5}+{x}_{16}}-0.3307\times {\text{cos}}\left({x}_{16}+2.175\right)\times \left({x}_{11}+{x}_{14}+0.7019\right)-\frac{0.008202\times {x}_{10}\times {x}_{14}}{\mathrm{cos }({x}_{8}+1.473)\times ({x}_{9}+{x}_{14})}-0.07444$$

Similarly, the RMSE, MARE and R^2^ parameters were calculated for this prediction model (Eq. 11). The RMSE, MARE and R^2^ parameters in the training data are 4.59, 0.77 and 0.09, and in the test data are 9.52, 1.26 and 0.0025, respectively (Table 4). Furthermore, this prediction model has failed in the hospital stay prediction of 167 out of 487 training data, and performed with an accuracy of 65.7%. Moreover, in test data, the achieved prediction model attain an accuracy of 61.25%, predicting the duration of hospital stay in 147 out of 240 data accurately (Table 3). Furthermore, sensitivity analysis revealed the high importance of PT, ESR, ALT, BUN, PTT and CRP in the hospital stay prediction of male COVID-19 patients (Fig. 4).

### Online prognosis prediction application

Based on the obtained prediction models, we have created an online application for prognosis prediction and triage of COVID-19 patients, which can be accessed on android, iOS mobile, and an online webpage. The application can be downloaded from the following link https://oaa.app.link/launch-app-a0f62d8f-9d33-481b-9f9b-7b33b016ce8d (registration for a free OpenAsApp account is required to access the application). In this application, at first, you select the gender, then by entering the routine hematological variables at the time of admission, you can forecast the duration of hospital stay, the admission to ICU, and the mortality using MGGP-based prediction models.

## Discussion

AI tools can aid physicians in the early diagnosis, and the triage of patients^23,24^. In addition, since COVID-19 can bring about from asymptomatic infection to severe disease and multi-organ damage, early stratification of those who may develop a severe disease has been a challenge for clinical physicians^25,26^. In this regard, the present study aimed to predict the prognosis of COVID-19 patients using MGGP and conventional laboratory tests at the time of admission. Furthermore, the significance of each parameter in the obtained estimation models is assessed. In this regard, the current study indicated that MGGP is capable of predicting the mortality outcome of COVID-19 patients with an accuracy of 60–92%. Furthermore, MGGP predicted the ICU admission of COVID-19 patients with an accuracy of 76–91%.

Similarly, the outcome prediction models of COVID-19 patients have been developed by various AI tools in previous studies^27,28^. For instance, in a study by Santos-Lozano et al.^29^, they exploited an artificial neural network (ANN) for the outcome prediction of COVID-19 patients using laboratory findings. The obtained prediction models performed with 85% accuracy in the training data and with 88% accuracy in the test data^29^. Moreover, Yao et al.^30^ predicted severe COVID-19 patients with the application of a support vector machine using clinical, hematological, and urinary findings. They estimated severe COVID-19 with an accuracy of about 81%. Lee et al.^31^ predicted ICU admission with an accuracy of about 78% and mortality with an accuracy of 84% using deep artificial neural networks and clinical findings, demographic characteristics, and hematological findings of the COVID-19 patients. In another study, Ustebay and colleagues^32^, applied eight different machine learning method such as Support vector machines (SVM), logistic regression, random forest, XGBoost, multilayer perceptron, extra trees, CatBoost, and k-nearest neighbors classifiers to predict the prognosis of COVID-19 patients, based on the clinical, demographic and laboratory data^32^. They demonstrated that extra tree and CatBoost classifiers achieved a higher accuracy than other machine learning methods. Also, they observed that C-reactive protein, the ratio of lymphocytes, lactic acid, and serum calcium had higher impacts on the prognosis prediction of COVID-19^32^. In another study, Booth et al.^12^, applied SVM to predict the mortality of COVID-19 patients. They observed that SVM could predict the mortality of COVID-19 patients with 91% sensitivity and 91% specificity based on the level of C—reactive protein, blood urea nitrogen, serum calcium, serum albumin, and lactic acid^12^. Compared to the previous studies, in the present study, in a bid to develop a triage model, the most common hematological tests at the time of hospitalization are exclusively used for developing the prediction models. Therefore, the achieved prediction models can be applied for the triage of COVID-19 patients in centers with limited facilities. Unarguably, by considering the clinical findings during the hospitalization, and more advanced laboratory tests, such as venous blood gas or interleukin levels, it is possible to provide more accurate models for the prognosis prediction of COVID-19 patients^31^. Furthermore, another strength of the present study is considering three outcomes including mortality, duration of hospital stay, and ICU admission as the prognosis of COVID-19 patients. Considering the fact that the present study aimed to develop a triage model for COVID-19 patients, compared to the previous studies, all developed models achieved promising accuracies.

In this study, MGGP predicted the length of hospital stay with an accuracy of 53–65%. Despite the importance of hospital stay in healthcare decision-making and resource allocation, limited studies have been conducted to predict the hospital stay of COVID-19 patients. In the current study, the accuracy of hospital stay prediction models was relatively lower than other predicted outcomes. It seems that in addition to the patient's clinical condition, various factors, such as the subjective clinical suspicion of the physician, and the number of available hospital beds at the time of the outbreak, impact the duration of hospital stays.

The advantage of MGGP over nonlinear regression models is that both the structure and parameters of a prediction model can be accomplished by MGGP. As a result, MGGP can develop a prediction model regardless of the nature of the problem, while the user can reconcile the accuracy and complexity of the prediction model by controlling the crucial MGGP parameters (i.e., G_max_ and d_max_)^17^. This means that although the obtained prediction models were acceptably accurate, developing more accurate prediction models can be achieved through increasing G_max_ and d_max_. However, this inevitably increases the complexity of the calculated prediction models^33^. Furthermore, since the evolution of each gene is independent, not only MGGP can deal with more complex problems, but also benefits from parallel computation, which results in the faster convergence and better scalability. In addition, in MGGP, each gene evolves for certain features of the data. This adaptability to the problem structure can result in better performance of developed models.

In addition, sensitivity analysis was conducted for the input variables of each prediction model. As shown in Figs. 2–4, the sensitivity analysis of ICU admission prediction models revealed the significant impact of age and AST. This suggests that these parameters may have the greatest effects on the ICU admission of COVID-19 patients. Also, regarding the duration of hospital stay, BUN and PT may have a greater impact on the prediction of hospital stay. Similarly, the sensitivity analysis of mortality prediction models revealed the importance of AST and BUN in the prediction of outcomes. Overall, BUN and AST may possess a greater impact on the prognosis prediction of COVID-19 patients. Similar to our findings, Liu and colleagues in a multicenter retrospective study on more than twelve thousand COVID-19 patients^34^, demonstrated that BUN level had a strong correlation with the adverse outcome of the COVID-19. They indicated that BUN level not only presents the renal dysfunction, but also reflect the inflammatory status, cardiac output, sepsis, and other adverse outcomes which had been reported to be associate with the pathogenesis of COVID-19 patients^34^. In this regard, the association of BUN level and COVID-19 prognosis has been demonstrated in several other studies as well^35,36^. Also, Wang an colleagues, in a meta-analysis, observed that there is strong correlation between the increased level of AST and COVID-19 mortality^37^. Similarly, Sharma et al.^38^, in another meta-analysis on 12,882 COVID-19 patients, demonstrated that increased level of AST was associated poor prognosis in COVID-19 patients^38^. It is hypothesized that the increased level of AST in COVID-19 patients may be multifactorial, reflecting the hepatocellular injury and muscular damage^39^. Nevertheless, further clinical studies considering the impact of AST and BUN level in the prognosis of COVID-19 patients is suggested.

There are several limitation to the present study. First, the present study is a single-center study from Iran. In this regard, based on the previous studies, the COVID-19 may have different impacts on various ethnics and races. Furthermore, the local guidelines inevitably may impact the diagnosis, treatment, and management of COVID-19 patients. Therefore, the present study may subjected to the institutional bias. Also, the developed models of the present study were only included routine hematologic variables at the time of admission, as mentioned earlier, taking more clinical, and demographic features may brought about more accurate prognosis prediction. Another limitation of the present study, is the impact of vaccination programs and emerge of new variants of SARS-CoV-2, which may bias the result of the present study. Finally, as an external validation of the present study, further multi-center studies with more sample size is encouraged.

## Conclusion

The present study demonstrated that MGGP is capable of predicting the prognosis of COVID-19 patients based on the routine hematological variables at the time of admission with promising accuracy. Therefore, MGGP based triage models could help identifying those who may develop a severe COVID-19 at the time of admission. In addition, the present study revealed that among the common hematological variables, the level of BUN and AST have a greater impact on the prognosis of COVID-19 patients. In this regard, further clinical studies considering the impact of AST and BUN level in the prognosis of COVID-19 patients is suggested. Furthermore, a simple prognosis prediction and triage application are developed, which can be accessed freely through https://oaa.app.link/launch-app-a0f62d8f-9d33-481b-9f9b-7b33b016ce8d. This smartphone and web-based application can be practically utilized for the triage of COVID-19 patients, while it can be improved with a larger dataset.

## Data Availability

The data that support the findings of this study are available on request from the corresponding author (HRN).

## References

[1] Danesh, F., Dastani, M. & Ghorbani, M. *Retrospective and Prospective Approaches of Coronavirus Publications in the Last Half-Century: A Latent Dirichlet Allocation Analysis* (Library Hi Tech, 2021).

[2] Hasan, M. K., Islam, M. M. & Hashem, M. Mathematical model development to detect breast cancer using multigene genetic programming. In *2016 5th International Conference on Informatics, Electronics and Vision (ICIEV)* (IEEE, 2016).

[3] *Coronavirus disease (COVID-19) Weekly Epidemiological Update*. 8 Nov 2021]; https://www.who.int/emergencies/diseases/novel-coronavirus-2019/situation-reports.

[4] Pascarella G (2020). COVID-19 diagnosis and management: A comprehensive review. J. Intern. Med..

[5] Karimi Shahri M, Niazkar HR, Rad F (2021). COVID-19 and hematology findings based on the current evidences: A puzzle with many missing pieces. Int. J. Lab. Hematol..

[6] Laventhal N (2020). The ethics of creating a resource allocation strategy during the COVID-19 pandemic. Pediatrics.

[7] Wynants L (2020). Prediction models for diagnosis and prognosis of covid-19: Systematic review and critical appraisal. Bmj.

[8] Elshazli RM (2020). Diagnostic and prognostic value of hematological and immunological markers in COVID-19 infection: A meta-analysis of 6320 patients. PLoS ONE.

[9] Terpos E (2020). Hematological findings and complications of COVID-19. Am. J. Hematol..

[10] Sattar M (2022). Lung cancer prediction using multi-gene genetic programming by selecting automatic features from amino acid sequences. Comput. Biol. Chem..

[11] Niazkar M, Niazkar HR (2020). Covid-19 outbreak: Application of multi-gene genetic programming to country-based prediction models. Electron. J. Gen. Med..

[12] Booth AL, Abels E, McCaffrey P (2021). Development of a prognostic model for mortality in COVID-19 infection using machine learning. Mod. Pathol..

[13] Zhao S (2020). Estimating the unreported number of novel coronavirus (2019-nCoV) cases in China in the first half of January 2020: A data-driven Modelling analysis of the early outbreak. J. Clin. Med..

[14] Koza JR (1994). Genetic Programming II.

[15] Kinnear KE (1994). Advances in Genetic Programming.

[16] Langdon WB, Poli R (2013). Foundations of Genetic Programming.

[17] Langdon W (2021). Genetic programming convergence. Genet. Program. Evol. Mach..

[18] Niazkar M (2023). Multigene genetic programming and its various applications. Handbook of Hydroinformatics.

[19] Santoso L (2020). A genetic programming approach to binary classification problem. EAI Endorsed Trans. Energy Web.

[20] Merzougui N, Djerou L (2021). Multi-gene genetic programming based predictive models for full-reference image quality assessment. J. Imaging Sci. Technol..

[21] Searson, D. *GPTIPS: Genetic Programming and Symbolic Regression for MATLAB* (2009).

[22] Zakwan M, Niazkar M (2021). A comparative analysis of data-driven empirical and artificial intelligence models for estimating infiltration rates. Complexity.

[23] Feng C (2021). A novel triage tool of artificial intelligence assisted diagnosis aid system for suspected COVID-19 pneumonia in fever clinics. MedRxiv.

[24] Qin ZZ (2021). Tuberculosis detection from chest x-rays for triaging in a high tuberculosis-burden setting: An evaluation of five artificial intelligence algorithms. Lancet Digit. Health.

[25] Feng C (2021). A novel artificial intelligence-assisted triage tool to aid in the diagnosis of suspected COVID-19 pneumonia cases in fever clinics. Ann. Transl. Med..

[26] Heldt FS (2021). Early risk assessment for COVID-19 patients from emergency department data using machine learning. Sci. Rep..

[27] Zhang S (2021). Identification and validation of prognostic factors in patients with COVID-19: A retrospective study based on artificial intelligence algorithms. J. Intensive Med..

[28] Xiao Y (2021). Machine learning discovery of distinguishing laboratory features for severity classification of COVID-19 patients. IET Cyber-Syst. Robot..

[29] Santos-Lozano A (2020). Can routine laboratory variables predict survival in COVID-19? An artificial neural network-based approach. Clin. Chem. Lab. Med..

[30] Yao H (2020). Severity detection for the coronavirus disease 2019 (COVID-19) patients using a machine learning model based on the blood and urine tests. Front. Cell Dev. Biol..

[31] Li X (2020). Deep learning prediction of likelihood of ICU admission and mortality in COVID-19 patients using clinical variables. PeerJ.

[32] Ustebay S (2023). A comparison of machine learning algorithms in predicting COVID-19 prognostics. Intern. Emerg. Med..

[33] Searson, D. P., Leahy, D. E. & Willis, M. J. GPTIPS: An open source genetic programming toolbox for multigene symbolic regression. In *Proceedings of the International Multiconference of Engineers and Computer Scientists* (Citeseer, 2010).

[34] Liu Y-M (2021). Kidney function indicators predict adverse outcomes of COVID-19. Medicine.

[35] Cheng A (2020). Diagnostic performance of initial blood urea nitrogen combined with D-dimer levels for predicting in-hospital mortality in COVID-19 patients. Int. J. Antimicrob. Agents.

[36] Ye B (2021). Association between an increase in blood urea nitrogen at 24 h and worse outcomes in COVID-19 pneumonia. Renal Fail..

[37] Wang Y (2021). An updated meta-analysis of AST and ALT levels and the mortality of COVID-19 patients. Am. J. Emerg. Med..

[38] Sharma A (2021). Liver disease and outcomes among COVID-19 hospitalized patients: A systematic review and meta-analysis. Ann. Hepatol..

[39] Aloisio E (2021). Sources and clinical significance of aspartate aminotransferase increases in COVID-19. Clin. Chim. Acta.

